# A quantitative model of relation between respiratory-related blood pressure fluctuations and the respiratory sinus arrhythmia

**DOI:** 10.1007/s11517-018-1939-4

**Published:** 2018-12-22

**Authors:** Teodor Buchner

**Affiliations:** Faculty of Physics, Warsaw University of Technology, ul. Koszykowa 75, 00-662 Warsaw, Poland

**Keywords:** Respiratory sinus arrhythmia, Heart rate control, Blood pressure, Mathematical model, Nonlinear dynamics

## Abstract

In order to propose an interpretation of recent experimental findings concerning short-term variability of arterial blood pressure (ABP), heart rate variability (HRV), and their dependence on body posture, we develop a qualitative dynamical model of the short-term cardiovascular variability at respiratory frequency (HF). It shows the respiratory-related blood pressure fluctuations in relation to the respiratory sinus arrhythmia (RSA). Results of the model-based analysis show that the observed phenomena may be interpreted as buffering of the respiratory-related ABP fluctuations by heart rate (HR) fluctuations, i.e., the respiratory sinus arrhythmia. A paradoxical enhancement (PE) of the fluctuations of the ABP in supine position, that was found in experiment, is explained on the ground of the model, as an ineffectiveness of control caused by the prolonged phase shift between the the peak of modulation of the pulmonary flow and the onset of stimulation of the heart. Such phasic changes were indeed observed in some other experimental conditions. Up to now, no other theoretical or physiological explanation of the PE effect exists, whereas further experiments were not performed due to technical problems. Better understanding of the short-term dynamics of blood pressure may improve medical diagnosis in cardiology and diseases which alter the functional state of the autonomous nervous system.

**Graphical Abstract:**
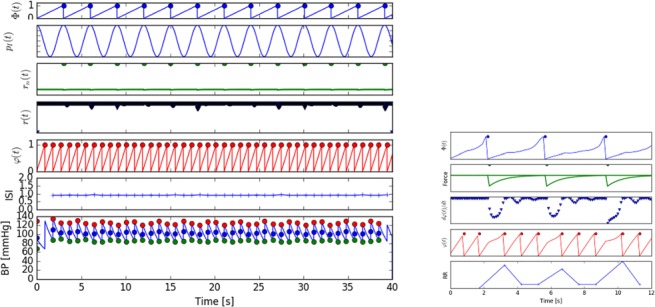
A simple mathematical model of cardiorespiratory dynamics. A novel class of mathematical models of blood pressure dynamics in humans allows to represent respiratory modulation of Arterial Blood Pressure. The model shows how the phase shift in neural control of the heart rate may produce Paradoxic Enhancement of respiratory Blood Pressure fluctuations. Observed in experiment. The model has many options for further development.

A simple mathematical model of cardiorespiratory dynamics. A novel class of mathematical models of blood pressure dynamics in humans allows to represent respiratory modulation of Arterial Blood Pressure. The model shows how the phase shift in neural control of the heart rate may produce Paradoxic Enhancement of respiratory Blood Pressure fluctuations. Observed in experiment. The model has many options for further development.

## Introduction

There are still many mysteries in cardiovascular regulation. Providing a solution to them not only pushes forward the frontier of science, but also enables us to formulate new methods of cardiological diagnosis and risk stratification, which results in more efficient patient handling - see [1] and [2] for recent successful examples of such an approach.

One of important physiological variables under consideration is the arterial blood pressure (ABP). Increased variability of ABP is related with increased risk of end-organ damage [3]. Hence, it is important to understand the mechanisms that introduce or reduce the instability of ABP. The relation between the heart rate (HR) and the ABP is a subject of intensive experimental and theoretical study (see, e.g., the review [4]). Since the advent of the frequency domain analysis of the cardiovascular regulation, the dynamics of HR and ABP in certain frequency regimes is often studied separately [5]. Here, we would like to concentrate entirely on the dynamics in high frequency band (HF), i.e., 0.15–0.5 Hz [6].

The primary physiological reason for the variability of ABP in the HF band is the change of diameter of the chest, related with respiration. The large vessels above the diaphragm, as well the heart itself, are exposed to the transmural action of the intrathoracic pressure. This pressure follows the respiration and modulates directly the left ventricular stroke volume (SV), which can be directly measured using Doppler techniques [7–9]. Recently, also, the respiratory modulation of the right SV was reported [10], with the quite unexpected phase shift to the left SV.

The transmural pressure imposes a mechanical modulation, which is unavoidable, and uncontrollable as such. Experiments such as Valsalva maneuver (forced exhalation against closed airway) reveal a complex action of physiological reflexes, which respond to the altered transmural pressure [11]. In pathological states, such as emphysema, this pressure may completely stop venous return during the Valsalva maneuver [12].

However, the mechanical modulation is not the only source of the ABP variability in the HF region. The heart exhibits large plasticity of the pumping efficiency, which is subject to many regulatory reflexes, including those, related to respiration. The phenomenon of the acceleration of the heart rate after inspiration, often termed respiratory sinus arrhythmia (RSA), is known since 1871 [13]; however, the debate on its central [14] or reflex origin [15] as well on its teleological role (as detailed below) is still open.

Concerning the dynamics of the HR, there seems to be a number of distinct mechanisms: central and peripheral, which affect its variability in the HF band. The RSA is primarily mediated by respiratory-related, efferent parasympathetic activity from vagal cardiac fibers [16–18] which may be formally split into two components: the tonic and the phasic. The tonic component is traditionally called the “vagal tone,” which expresses its tonic (i.e., constant) character. Indeed, it responds to changes of mean ABP [21] by reducing the mean HR [19], which also equilibrates the alveolar ventilation with the pulmonary blood flow [20]. The phasic component is variable and related with the phase of respiration [22]. It reaches its maximum at the certain phase of respiration [22] and acts on the sinus node, which induces the RSA. Both effects, the tonic and the phasic, may be temporarily blocked by the bilateral cooling of the vagus nerve [19]. The phasic component is also modulated by the baroreflex [17].

The baroreflex is traditionally attributed to the sympathetic arm of the autonomous nervous system (ANS), as the manifestation of the effector arm of this reflex is most often observed in sympathetic activity: muscle sympathetic nerve activity (MSNA) [24] and preganglionic sympathetic activity [22]. It is well known, however, that the vagal component of efferent baroreflex arm is at least equally important [17]. The baroreflex, in particular [25], and the sympathetic activity in general [23], have many (nonlinear) relations to the RSA. When the total power of HRV was studied as a function of the voluntary breathing frequency, application of beta-blockers has revealed a rich nonlinear structure of this plot [23]. The chronotropic response to baroreceptor stimulus was shown to depend on the time from the last inspiration [27],[28]: hence, an important nonlinearity of this response was revealed.

The relation between the baroreflex and breathing is bidirectional: the respiratory modulation (gating) is present in sympathetic activity [22, 24], while on the other hand, sympathetic drive, including the baroreflex part, serves as an input for the pre-Bötzinger complex [26].

The nonlinearity, mentioned above, is sometimes modeled as a reciprocal relation between sympathetic and parasympathetic activity—with a nonlinear phase response function [29, 30]. This approach may be disputable, as the assumption of reciprocity is based merely on the general principle of homeostasis [56]. The physiological reality is much more complex—see, e.g., [31] for a huge cellular level mathematical model of a single sinus node cell, to say nothing about the collective behaviour thereof [32].

Finally, the RSA was also observed in denervated hearts [33] indicating a contribution from mechanical coupling via stretch receptors in the wall of the right atrium, where the sinus node (natural primary heart pacemaker) is situated, i.e., the Bainbridge reflex.

Despite many attempts, there is no general consensus about the teleological role of the RSA, however there seems to be at least an agreement, that it has some purpose, and it is not some unwanted byproduct. There are three major hypotheses concerning the physiological purpose of RSA: (1) improvement of gas exchange [34], (2) minimization of the energy consumption for the heart [35], and (3) reduction of the ABP fluctuations [10, 36]. These functions are not mutually exclusive [10]; however, it remains interesting, which physiological regulatory loop has the largest potential. The determination, which physiological variable (e.g., pCO2 or ABP) is more potent, from the perspective of the autonomous nervous system (ANS), may have important consequences for medical diagnosis and therapy. Interestingly, the hypothesis concerning the possible role of RSA in enhancement of pulmonary gas exchange [34] has been questioned by recent research [38], which means, that unless some new evidence appears, only the hypotheses (2) and (3) remain unfalsified for the time being. It is out of the scope of current paper to give a definitive answer.

The hypothesis that the role of the RSA is to reduce the ABP fluctuations is based on two premises. The first premise is the fact that the phase relations between the ABP and the HR show that they are nearly in counter-phase. This can be measured using spectral domain [9, 10, 37] or using correlation and timing of the response to nonstationarities [61]. The second premise is the fact that the suppression of RSA generally leads to enhancement of the ABP fluctuations in the HF band [9, 36].

The premises for the validity of the buffering hypothesis seem quite convincing; however, there is one interesting and yet unexplained finding, which presents itself as an apparent obstacle. A few distinct research groups using different research protocols [9, 37, 39, 52, 59] have reported, that the hypothetic buffering mechanism exhibits unexpected properties. Not only was the buffering dependent on the body position [52], but also it seemed to act differently on different signals derived from the ABP waveform [9, 37, 39, 59]. In the supine position of the body, only the fluctuations of the mean arterial pressure (MAP) were buffered by the presence of the RSA, whereas the fluctuations of the systolic arterial pressure (SAP) were actually paradoxically enhanced. Coming to teleology, the enhancement of respiratory-related fluctuations of the ABP does not seem to have an apparent physiological role. Therefore, we rather interpret the mentioned enhancement at least as an intriguing inefficiency of the control mechanism, if not as a proof, that the alleged buffering is only a by-product of the cardiorespiratory regulation, which rather serves some other physiological purposes.

Unfortunately, research groups which performed some of the recent experiments [9, 36] confirmed that the experiments were not conducted in the upright position:^1^ either due to technical problems with the assessment of stroke volume in the upright position [9] or due to the fact, that it was out of the scope of the research protocol [36], which was not neutral, as it included pharmaceutical blockade of autonomous reflexes. Therefore experimental study on this effect remains technically quite cumbersome.

The purpose of this paper is to present a model-based analysis of the possible mechanism underlying the phenomenon of selective buffering, understood as the dependence of the buffering of the ABP-related variables on the position of the body, and the related paradoxical enhancement of systolic BP fluctuations.

## Methods

The mechanism of selective buffering is studied using a simple mathematical model of cardiovascular dynamics, consisting of three compartments, with nonlinear elements. Minimalistic models of this type were proposed by Arthur Guyton. Guyton calculated the mean circulatory filling pressure and introduced the term “venous return” [40] on the ground of the model, in which the whole cardiovascular system was reduced to only two segments. Note, also, that the model is quantitative: its role is not to mimic the exact waveform of the ABP wave, as there seem to be much better models serving this purpose [46]. Its purpose is to describe the impact of phase, in which the neural control is delivered, on the ABP-related variables, in order to provide a theoretical explanation of the yet unexplained experimental facts.

The model describes the dynamics of the blood pressure in three different compartments: the arterial compartment (AC), the cardiopulmonary compartment (CC) and the venous compartment (VC). The glossary of parameters used in the model with their nominal values is given in Table 1.

**Table 1:** Glossary of model parameters and their nominal values

Symbol	Quantity	Nominal value
*C*_*a*_	Systemic arterial compliance	1.6 ml/mmHg
*C*_*v*_	Systemic venous compliance	100 ml/mmHg
*C*_*c*_	Effective cardiopulmonary compliance	4.3 ml/mmHg
*Z*_*vc*_	Ventricular outflow conductance (CO)	200 ml/(s mmHg)
*Z*_*av*_	Arterial outflow conductance (1/TPR)	1.1 ml/(s mmHg)
*Z*_*ca*_	Effective cardiopulm. outflow conduct.	166 ml/(s mmHg)
*p*_0_	Intrathoracic pressure	–6.6 mmHg
*p*_1_	Intrathoracic pressure amplitude	1.5 mmHg
*r*	Resting heart rate	66 BPM
*r*_0_	Neural control amplitude	1s
*τ*	Neural control delay	0.3 s
*τ*_*n*_	Neural control decay rate	0.3 s
*R*	Resting respiratory rate	20 BPM

The core of the model is constituted by the set of ordinary differential equations for blood pressure in all three compartments: *p*_*a*_ in arterial - (1), *p*_*c*_ in cardiopulmonary - (2) and *p*_*v*_ in venous compartment - (3):

1 $$\begin{array}{@{}rcl@{}} C_{a} \frac{dp_{a}}{dt}\!&=&\! \displaystyle\sum\limits_{i} \delta (t - t_{i}) Z_{ca} (p_{c}-p_{I}) - Z_{av} p_{av} \end{array} $$

2 $$\begin{array}{@{}rcl@{}} C_{c} \frac{dp_{c}}{dt}\!&=&\!max(Z_{vc}\!\cdot\! p_{vc},0) - \displaystyle\sum\limits_{i} \delta (t - t_{i}) Z_{ca} (p_{c} - p_{I}) \end{array} $$

3 $$\begin{array}{@{}rcl@{}} C_{v} \frac{dp_{v}}{dt}\!&=&\! Z_{av} p_{av} - max(Z_{vc}\cdot p_{vc},0) \end{array} $$

The pressures with double indices represent blood pressure gradients between neighboring compartments (e.g., *p*_*av*_ = *p*_*a*_ − *p*_*v*_). Each compartment is characterized by two lumped parameters: the compliance and the outflow conductance (the reciprocal of the resistance). *Z*_*xy*_ denotes the conductance between the compartments *x* and *y*, and *p*_*I*_ stands for intrathoracic pressure. The inertial effects due to blood flow are neglected, following [41]. The normative values of parameters listed in Table 1, apart from those that characterize the neural control, were taken from [41].

**Fig. 1:**
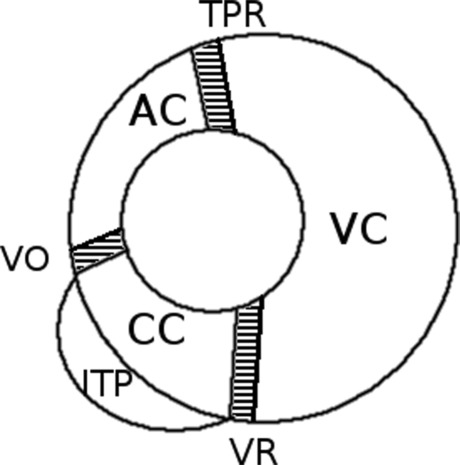
Model setup. Cardiopulmonary, arterial and venous compartment are denoted as CC, AC and VC respectively. The flows between the compartments are: venous return (VR), ventricular outflow (VO) related with cardiac output (CO), and flow through the total peripheral resistance (TPR). The transmural pressure acting on the cardiopulmonary compartment is the intrathoracic pressure (ITP)

The mathematical form of (1) and (2) resembles the “kicked” oscillator models which where extensively studied in nonlinear dynamics [43] and may be converted to discrete time systems, such as described in ref. [44] or [45]. The heart beats take place at times *t*_*i*_, and at these times the pressures become ‘kicked’. The mathematical process, which defines the timeseries *t*_*i*_ is described further in text. The model was implemented in Matlab, with the help of the RCVSIM framework [42], rewritten in C#, and finally in Python [62]. Both implementations gave quantitatively equivalent results. The structural stability of the model was verified by varying the parameters by ± 10*%* about their nominal values: the HR and the ABP for all parameter values were within the physiological limits. The model setup is shown in Fig. 1.

### The cardiopulmonary compartment

Introduction of the cardiopulmonary compartment, as defined in Eq. 2, is a novelty, which was not considered in [40, 41] or any models referenced therein. This compartment represents the function and properties of the heart, the valves and the large vessels situated in the thorax. The compartment is a surrogate, which allows to treat the dynamics within the pulmonary circulation and the heart as a single entity. From the point of view of detailed models, such as the model by Olufsen *et al.* [46], the model presented here is a crude approximation, but gives a computationally inexpensive mathematical description of the mechanical modulation, imposed on blood flow by the respiratory effort, which is sufficient for our purpose. The inflow into the cardiopulmonary compartment is represented by a conductance *Z*_*vc*_, which might be altered, for instance, to model orthostatic changes of the venous return.

As the cardiopulmonary compartment is anatomically located in the thorax, the external (transmural) pressure which acts on the vessels located within this compartment is the intrathoracic pressure *p*_*I*_. It sets the reference pressure that affects the outflow from this compartment. Such approach is used, e.g., in cerebrospinal fluid (CSF) models [47], where the ABP counteracts the intracranial pressure. The intrathoracic pressure *p*_*I*_ changes in phase with breathing, as defined by Eq. 4:

4 $$ p_{I}(t)=p_{0}+p_{1}\left( 1 + \cos 2 \pi {\Phi}(t)\right) $$

In Eq. 4, the depth of respiratory-related modulation is considered to be constant as the first order of approximation.

Following [41], the action of valves is mimicked by an ideal diode, represented by the *max* function in Eqs. 2 and 3 which prevents the backward flow. Introduction of the diode introduces nonlinearity into the equations. Of course real valves do not have ideal characteristics, however definitely they break the symmetry with respect to the direction of the flow.

### Modeling the hemodynamic effect of a heart action

The popular relation ABP = CO x TPR does not consider the impulse nature of the heart action. Our model introduces the heart as an impulse pump. The heart phase is modeled using the “integrate and fire” type of model [48]. Following the idea of Guevara and Glass [49] and of Seidel and Herzel [29], we introduce a single phase variable *φ*(*t*) with the range [0,*∞*], which represents the phase of the cardiac cycle:

5 $$ \frac{d\varphi}{dt}=r+\displaystyle\sum\limits_{j} f\left( t - t_{j} - \tau\right) \cdot F\left( \varphi \bmod{1}\right) $$

Functions *f* and *F* in Eq. 5 represent the neural control of the heart rate and the sum is over all breaths, as explained in the next paragraph. For convenience, the phase *φ*(*t*) is normalized to 1 instead of 2*π* (as in term *φ* mod 1 where phase is limited to the [0,1] range). The heartbeats are generated at times *t*_*i*_ whenever the value of the phase is integer:

6 $$ t_{i}: \varphi(t_{i})=i $$

The hemodynamic effect of heart contraction is a short, instantaneous flow pulse in Eqs. 1 and 2. We found such a flow to be qualitatively similar to that obtained from the model of varying elastance [41]. It is worth noting, that the derivative of the varying elastance in a functional form used by Mukkamala [41] undoubtedly resembles the Dirac delta function. In consequence this type of model has a form of a “kicked Windkessel.”

The Frank-Starling law is not directly introduced into the model. The effect of the cardiac filling time is, however, present in the model, as the *p*_*c*_ changes nearly reciprocally to *p*_*a*_ (c.f. Fig. 3). If the RR interval is short and the *p*_*a*_ at the end of diastole is still high, the BP in the cardiopulmonary compartment *p*_*c*_ will be low due to the incomplete filling. In consequence, the next stroke volume (second term on the r.h.s. of Eq. 2) will be reduced. This argument holds as long as the fluctuations of the heart rate are not large.

The stroke volume in our model, represented by *Z*_*ca*_, does not depend directly on the afterload, represented by *p*_*a*_. The only dependence is indirect: through the cardiac filling time, as explained above. The value of *p*_*I*_ in Eq. 4 is modulated by breathing. The consequence is a direct modulation of the cardiopulmonary outflow (i.e., the stroke volume [8]) and hence the modulation of the pulse pressure (PP) and the SAP with the frequency of respiration.

Note, that for brevity, we do not introduce any phase shift between the respiration and the modulation of the cardiac outflow; however, presence of this phase shift will be mimicked by other variables.

### The respiratory rhythm and neural control of the heart rate

The respiratory rhythm is introduced as another “integrate and fire” oscillator, with the dynamics of its phase variable Φ(*t*) described by Eq. 7:

7 $$ \frac{d{\Phi}}{dt}=R $$

The respiratory rate *R* is constant, however modulation may be introduced easily (c.f. [30]). The firing times for breathing are denoted by *t*_*j*_ .

8 $$ t_{j}: {\Phi}(t_{j})=j $$

The neural feedback considered in the model is the respiratory-related neural activity (second term in Eq. 5) which synchronizes the heart phase with the phase of respiration. Intuitively, we treat it as vagal, but as discussed in the preceding chapter, it includes all types of respiratory-related neural activity, that acts on the heart: not necessarily vagal, and even not necessarily neural (c.f. [33]).

As the effect of vagal activity on the cardiac pacemaker was shown to be phase-dependent [16, 50], and the same effect was observed for baroreflex [27], to account for this fact, we use the phase effectiveness function in the semi-empirical form defined by Eq. 9, as defined by Seidel and Herzel [29]:

9 $$ F(\varphi) = \varphi^{1.3}(\varphi-0.45)+\frac{(1-\varphi)^{3}}{(1-0.8)^{3}+(1-\varphi)^{3}} $$

**Fig. 2:**
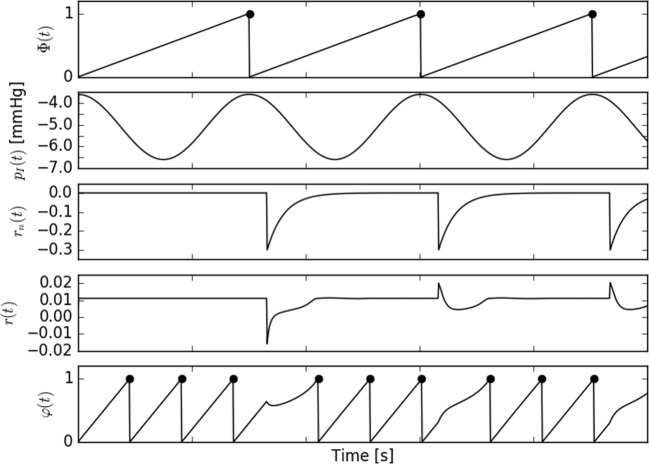
Schematic of the neural input in the kicked Windkessel model. From the top there are: respiratory phase Φ(*t*), intrathoracic pressure (peak of inspiration related to the minimum of *p*_*I*_. Zero phase at onset of inspiration, where the *p*_*I*_ attains the maximum. Third curve, marked as *r*_*n*_ shows a single kick, as defined by Eq. 10. Kicks are delivered at a short delay *τ* after each reset of Φ (bemark a small phase shift between the Φ and the *r*_*n*_). Fourth panel shows the r.h.s of Eq. 5, i.e., the cumulated effect of neural input, acting through the phase sensitive curve (9). The phase velocity *r*(*t*) is either increased (like in breath 2 and 3) or decreased (like in breath 1). As the decay of the neural force is slow, the net effect on phase velocity *r* may be bipolar. Bottom row shows the resulting heart phase *φ*(*t*)

The neural input in this model is simplified: it is added to the cardiac phase as a spike, with a subsequent exponential decay, at time *τ* after each inspiration onset (Fig. 2), Eq. 5. The magnitude of its action depends on the time of arrival within the cardiac cycle, similarly to the experimental evidence [16, 17]. The appearance of the spike is referred to as “application of control.” Note, that the respiratory rhythm is slower than the HR; thus, the control is applied approximately every third heart beat, which is well visible in Fig. 2.

The cardiac oscillator responds by a lengthening or a paradoxical shortening (“vagal paradox” [16]) of the cardiac cycle, depending on the sign of the function *F*(*φ*) for the value of the cardiac phase *φ* at the time when the control is applied.

The mathematical form of such a decaying spike is described by Eq. 10

10 $$ f(t)=r_{o} \cdot {\Theta}(t) \cdot e^{-{t}/{\tau_{n}}} $$

The exponential decay is multiplied by the Heaviside theta function to ensure that the activity is nonzero only for positive time (c.f. Eq. 5). The characteristic time *τ*_*n*_ for the decay process is long (0.3 s), which is justified by the fact, that vagal discharge was shown to have an after-effect, which could last even to second and third heart evolution after stimulation [16]. The neural control of the heart rate has two parameters: the amplitude of the spike *r*_0_ and the delay time *τ* (with respect to the inspiration onset) at which it is delivered. As mentioned above, this delay time is set under an assumption, that there is no phase shift between respiration and the cardiac outflow modulation. Of course in experiment some phase shift exists [10], but the phase set point may be chosen arbitrarily.

It is important to note, that the amplitude of control *r*_0_ is not anyhow related to the ABP. Here, we treat it as a free parameter, which will be discussed below.

## Results

**Fig. 3:**
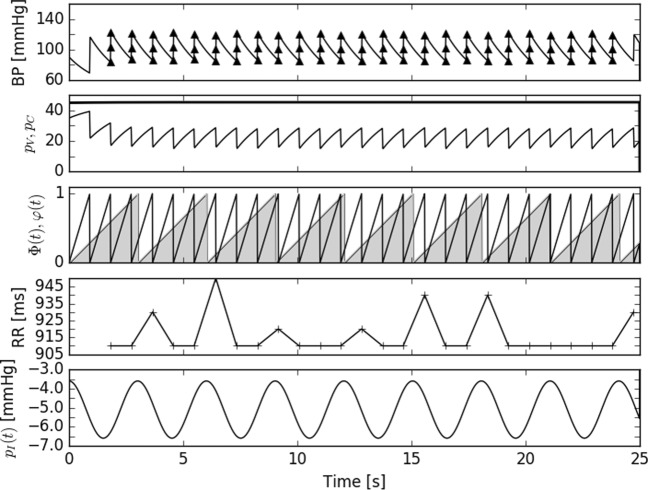
Example of numerical output of the model. The first row shows the arterial blood pressure *p*_*a*_. The second row shows pressures in venous compartment *p*_*v*_ (thick curve), and cardiopulmonary compartment *p*_*c*_ (thin curve). Blood pressure in the venous compartment is constant in this scale of the plot. The third row shows the phase of the heart *φ*(*t*) mod 1 (line) and the phase of the respiratory rhythm Φ(*t*) mod 1 (area curve). Control is applied at time *τ* after each maximum of Φ(*t*) mod 1. The fourth row shows the resulting RR interval variability, which is seemingly in-phase with the intrathoracic pressure *p*_*I*_, shown in the fifth row

The pressure waveforms generated with use of the model are shown in Fig. 3. The waveform of *p*_*a*_ follows the Windkessel principle: i.e., after each flow pulse (“kick”) there is an outflow to the venous compartment, proportional to the pressure gradient *p*_*av*_.

The magnitude of *p*_*a*_ is within physiological limits. The respiratory-related modulation of pulse pressure is visible as the *p*_*a*_ wave envelope modulation - c.f. Fig. 3a. The pressure in the venous compartment *p*_*v*_ is nearly constant because of the venous compliance *C*_*v*_ which is two orders of magnitude larger than the compliances of the other two compartments. The value of *p*_*v*_ is slightly above the physiological level, which seems justified given the qualitative character of the model. Blood pressure in the cardiopulmonary compartment *p*_*c*_ exhibits decreases seemingly correlated with cardiac outflow. During the diastole, *p*_*c*_ slowly increases due to the inflow from the venous compartment and during the systole suddenly decreases, due to momentary spike of outflow. The *p*_*c*_ does not have any direct bearing in the cardiovascular system, due to the fact, that this component describes all the cardiopulmonary circulation as one variable; however, it retains such physiological fact that the stroke volume depends on the time of the preceding diastole. The bottom traces in Fig. 3c show the respiratory phase and the cardiac phase, and the result of their interplay: i.e., the tachogram synchronized with the intrathoracic pressure *p*_*I*_.

### Effectiveness of buffering

**Fig. 4:**
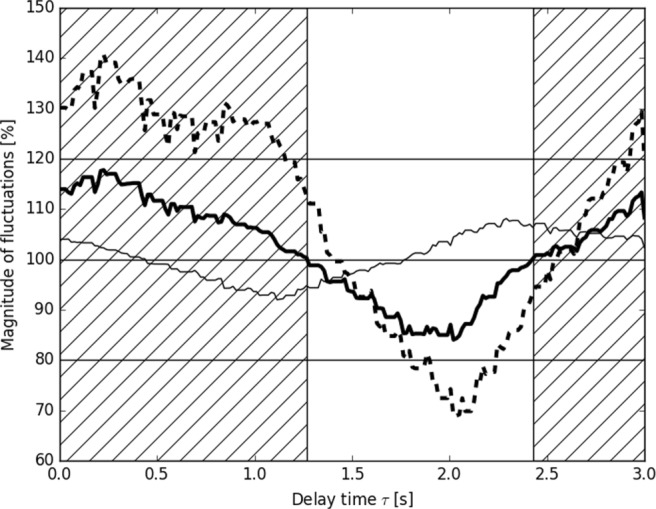
Magnitude of the fluctuations of BP as function of the delay time *τ*. Three ABP-derived variables are shown: the MAP (thick solid curve), the SAP (thin curve) and the DAP (thick dashed curve). Hatched area mark the ranges of ineffective control of the MAP

**Fig. 5:**
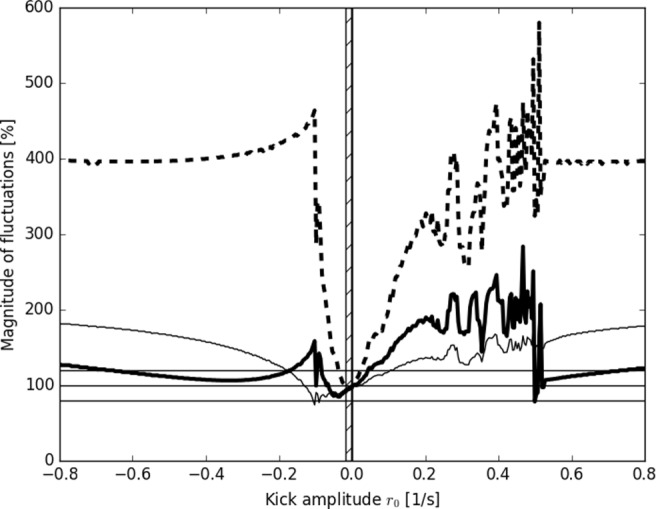
Magnitude of the fluctuations of BP as a function of the control amplitude *r*_0_. Three ABP-related variables are shown: MAP (thick solid curve), SAP (thin curve) and DAP (thick dashed curve). Hathed area marks ranges of an ineffective control of the DAP

In order to study the effectiveness of the postulated buffering, the parameter space of the model was explored. The two parameters that describe the neural control are as follows: the amplitude of the stimulus *r*_0_ and the delay *τ*. The effectiveness of the buffering as a function of each of these parameters is shown in Figs. 4 and 5, respectively. Note, that other model parameters, e.g., decay rate *τ*_*n*_ or resting rates for the respiration and the heart could be varied as well: despite its simplicity, the model is rich, and may be easily developed to serve various physiological purposes.

The effectiveness of buffering is assessed using the relative magnitude of fluctuations, as defined below. First, the characteristic values of arterial blood pressure *p*_*a*_ are determined for each heart beat: the diastolic (DAP), systolic (SAP) and mean (MAP) blood pressure. The MAP is calculated from the full waveform: between consecutive systoles.

Then, for each of variables, the reference value of the standard deviation of each of the blood pressure signals is determined: in the absence of the neural control (*r*_0_ = 0) but with the respiratory modulation present. As there is only one source of fluctuations in the system (the respiration) and a single buffering mechanism, there is no need to separate the power of fluctuations into different frequency bands. Therefore, standard deviation, equivalent to square root of total power of fluctuations, seems to be a sufficient measure to assess the buffering effectiveness. Next, for each value of the control parameter, the model is iterated for 40 seconds with the neural control turned on. From the model output, the standard deviation of each of the the ABP-related variables is calculated again, then divided by their reference values and expressed in percent. This procedure gives a relative magnitude of ABP fluctuations. If the magnitude of fluctuations is below 100%, we state, that for such a value of a parameter the control is effective. If the magnitude exceeds 100%, we state the control is ineffective as it paradoxically enhances the fluctuations instead of buffering them.

In Fig. 4, it may be seen that the range of effective control for each of the ABP-related variables is different. The MAP, is effectively buffered when *τ* is in the range [1.27, 2.43]*s*. In the middle of this range, approximately at *τ*_*c*_ = 1.7*s*, the range of effective control for the SAP ends. Below this critical value, the control is effective for both the SAP and the MAP, i.e., the fluctuations of both of them will be buffered. Above this threshold, only the MAP is effectively controlled. The DAP has quite a different range of effective control. It starts for delay times below *τ*_*c*_ and ends above the right extreme value yielding an effective control for the SAP.

Figure 5 shows that with respect to the amplitude of the control *r*_0_, for each controlled variable there exists a single minimum of the fluctuations. The regions of effective controls of all variables only partially overlap, and the most narrow range of control is the one for DAP, located at *r*_0_ ∈ [− 0.002, − 0.018], marked by hatched area in Fig. 5. Note, that due to the fact, that the neural control influences the phase velocity and not the phase itself, it is quite permissive to many dynamical phenomena, such as reversed direction of kick shown in Fig. 2. Hence, the behavior in *r*_0_ parameter space is rich, especially in the region of *r*_0_ > 0. The structures visible in this region are volatile and sensitive to the values of other parameters. For negative force *r*_0_ < 0, the structure is stable, being mainly rescaled and repositioned, while other parameters are varied.

## Discussion

The most important result to discuss is the selective control of the arterial blood pressure. It might be seen that, on the ground of the model, the control acts differently at each of the ABP-related variables. There exists a range of delay times in which the control is effective only for the MAP and not for the SAP. Such a selective control of blood pressure in the HF range was indeed observed in experiment. For instance in ref. [51] and ref. [52] the effect of a total cardiac autonomic blockade was discussed. It was observed that the blockade acts mainly on the fluctuations of the diastolic BP, leaving the fluctuations in the systolic BP quite unaltered. The popular approximate empirical formula for the mean arterial pressure: MAP = (2 DAP + SAP)/3 has a simple consequence: for the stabilization of the MAP it is two times more important to stabilize the diastolic ABP than the systolic ABP. Therefore, it is possible, that within a certain parameter range, the fluctuations of the systolic ABP will be enhanced and those of the mean ABP—reduced —due to the action of the same control mechanism. The destabilization of the SAP would then appear as a by-product of the control of fluctuations of the MAP. As the pressure fluctuations are not large, in the limit of long time the baroreflex signal may well be constant, which seems to justify (in the physiological setup discussed here) the constant value of *r*_0_ parameter in Eq. 10. Note e.g., that the denervation of the carotid (but not the aortic) baroreceptors does not alter the HF band of the ABP in rats [58].

Another experimental result that may be quite reliably explained using current theory is the presence of positional changes in the ABP variability amplitude, discussed in ref. [9] if we assume that the delay time between the ABP modulation and the application of control, represented by the parameter *τ* in eq. (5), depends on the position of the body. Actually, both the RSA pattern and the relative phase between the ABP and the HR depend on the position of the body [10, 37, 39, 53]. With the change of the body position not only the RSA pattern would change, but also the whole pattern would be shifted by 1 s with respect to the inspiration onset. This is equivalent to the change of the delay time *τ*. The explanation of the exact physiological mechanism that causes the change of the RSA pattern observed in [53] and phasic changes in cardiac stroke volume [10] remains unknown. One may only hypothesize that it originates from the positional change of the vertical distribution of the pulmonary blood flow. Such a change could introduce a phase shift between the phase of breathing and the phase of modulation of blood pressure in the cardiorespiratory compartment. On the ground of our model, we may consider this phase shift to be included in the delay time *τ*. Altered phasic relations were recently observed after application of the intermittent positive pressure ventilation (IPPV) [9]: note that the hydrostatic pressure within the thorax also changes with the position of the body.

The fact that the introduction of delay into the control loop may have a destabilizing effect was already studied theoretically [29, 30, 54]. The mechanism of instability, which appeared there, was a delay-dependent supercritical Hopf bifurcation, which destabilizes a fixed point (i.e., constant) solution and introduces oscillations with a frequency related to the delay length. The mechanism of the delay-induced instability, which we present here, is actually much simpler and reinforces the actually existing frequency by dephasing the control.

Note, however, that the role of the delay in physiological system and the oscillations it introduces may as well be constructive, and not disruptive, as in examples presented above. Examples in the area of systemic blood pressure control include models of Kitney [63] and Signorini et al. [64]. In nonlinear dynamics, a notable effect is the Pyragas chaos control method, in which a feedback loop, with suitable delay, stabilizes previously unstable pediodic orbits of a nonlinear dynamical system [65]. Stability of a delayed system is also an interesting problem in itself [66].

In the above discussion, we considered the ABP fluctuations at respiratory frequency as a by-product of the respiration. In fact, the same argumentation may be applied to the low frequency (LF) range and to the ABP fluctuations that appear there. Also in this range, the activity of HR and ABP are known to be related [55, 57], which may be interpreted as the effect of buffering. This opens the question whether the LF heart rate fluctuations may also have a destabilizing role under certain circumstances such as, e.g., the increased circulation time.

The model presented here is much less detailed than other compartmental models; however, it seems to fill the gap between detailed multi-compartment models of circulation which often have simplified heart rate control [46] and models presenting very detailed heart rate control, in which the circulatory part is largely reduced [29, 30].

The model mimics the physiological reality on basis of very modest assumptions. Further validation would require a direct measurement of phase delay in pulmonary circulation, related to postural change. If such a delay is indeed reported and directly measured, and the values are comparable to reported in this paper, the explanation proposed on the ground of this model will be proved experimentally. Further possibilities to extend this model, in order to mimic other physiological phenomena, and provide a putative explanation, have been mentioned in text.

## Conclusion

A simple quantitative model of cardiovascular variability was proposed. It reveals a possible mechanism of the selective buffering of the respiratory-related changes of ABP. On the ground of the model it is possible to explain the paradoxical enhancement of the ABP fluctuations, subject to changes of the position of the body, observed in experiment, as a result of an ineffective control, which come from altered phasic relations, which were also observed in various experimental conditions. The model bridges the models with the sophisticated neural control and the compartmental models. The model may be further extended: towards more realistic neural control or towards more refined compartment selection.

## Footnotes

1 M.Elstad, C.O.Tan, priv comm.
